# Acute exacerbation of ocular graft-versus-host disease and anterior uveitis after COVID-19 vaccination

**DOI:** 10.1186/s12886-023-03103-z

**Published:** 2023-08-18

**Authors:** Chen-Yu Lin, Hung-Jen Chien

**Affiliations:** Department of Ophthalmology, Taichung Veterans General Hospital, 1650 Taiwan Boulevard Sect. 4, Taichung, Taiwan

**Keywords:** Hematopoietic stem cell transplantation, Graft-versus-host disease (GVHD), Anterior uveitis, Severe acute respiratory syndrome coronavirus 2 (SARS-CoV-2), Coronavirus disease 2019 (COVID-19) vaccination

## Abstract

**Background:**

To report a case of simultaneous occurrence of acute exacerbation of ocular graft-versus-host disease (GVHD) and anterior uveitis following coronavirus disease 2019 (COVID-19) vaccination.

**Case presentation:**

A 60-year-old man with primary myelofibrosis and GVHD after receiving allogeneic hematopoietic stem cell transplantation (HSCT), developed acute exacerbation of ocular GVHD and anterior uveitis after receiving first dose of COVID-19 vaccine. The patient developed erythema of the eyelids, conjunctival hyperemia, superficial punctate keratopathy, and prominent anterior chamber inflammation in both eyes. The ocular GVHD and anterior uveitis were managed with mainly topical corticosteroids, antibiotics, lubricants, and systemic corticosteroids, but were difficult to control. Intravitreal injection of dexamethasone was administered, and the inflammation gradually subsided 6 months after the onset of initial symptoms.

**Conclusions:**

Clinicians should be aware of rare refractory anterior uveitis and acute exacerbation of ocular GVHD after COVID-19 vaccination in patients undergoing HSCT. Early diagnosis and aggressive treatment should be considered to reduce the likelihood of severe complications.

## Background

Graft-versus-host disease (GVHD) is a common complication of allogeneic hematopoietic stem cell transplantation (HSCT). Grafted immune cells attack the host tissue, including the skin, mouth, eyes, lungs, liver, gastrointestinal tract, genitalia, hematopoietic system and immune systems [1]. GVHD can be classified as acute or chronic, and most ocular complications occur in chronic GVHD. Cicatricial conjunctivitis and keratoconjunctivitis sicca are the main manifestations of chronic GVHD. Late complications include limbal stem cell deficiency and corneal scarring.

Because of the coronavirus disease 2019 (COVID-19) pandemic, over half of the world’s population has received at least one dose of vaccine to prevent morbidity and mortality. Two mRNA vaccines, the Pfizer-BioNTech BNT162b2 vaccine and Moderna mRNA-1273 vaccine received Emergency Use Authorization (EUA) from the US Food and Drug Administration (FDA) for the prevention of COVID-19 [2, 3]. However, the safety and efficacy of these vaccines in patients after allogeneic HSCT are still under investigation [4]. With regard to the sequelae of COVID-19 vaccination, Charlotte reported the classification and rates of uveitis, and Dat Ngo specifically discussed GVHD [5, 6].

Here, we present a case of simultaneous occurrence of acute exacerbation of ocular GVHD and anterior uveitis following Moderna mRNA-1273 vaccination.

## Case presentation

A 60-year-old male presented with acute onset of bilateral blurred vision, pain, and redness 3 days after receiving a first dose of Moderna mRNA-1273 vaccine. He was diagnosed with primary myelofibrosis and underwent allogeneic HSCT in 2014. Grade IV acute GVHD and subsequent chronic GVHD, which manifested mainly as skin and ocular lesions were diagnosed. The disease activity of GVHD became stable after treatment with systemic corticosteroids and immunosuppressants. All corticosteroids and immunosuppressants have been discontinued since 2019. Since then, his ocular condition has become stable. Moderate corneal opacity and band keratopathy in the right eye were noted; severe corneal opacity and pannus were noted in the left eye, with baseline intraocular pressure of approximately 18 to 20 mmHg in both eyes, a visual acuity of 20/100 in the right eye and hand motion in the left eye. The patient was treated for keratoconjunctivitis sicca with artificial tears. In addition, no previous attack of uveitis was recorded.

**Fig. 1:**
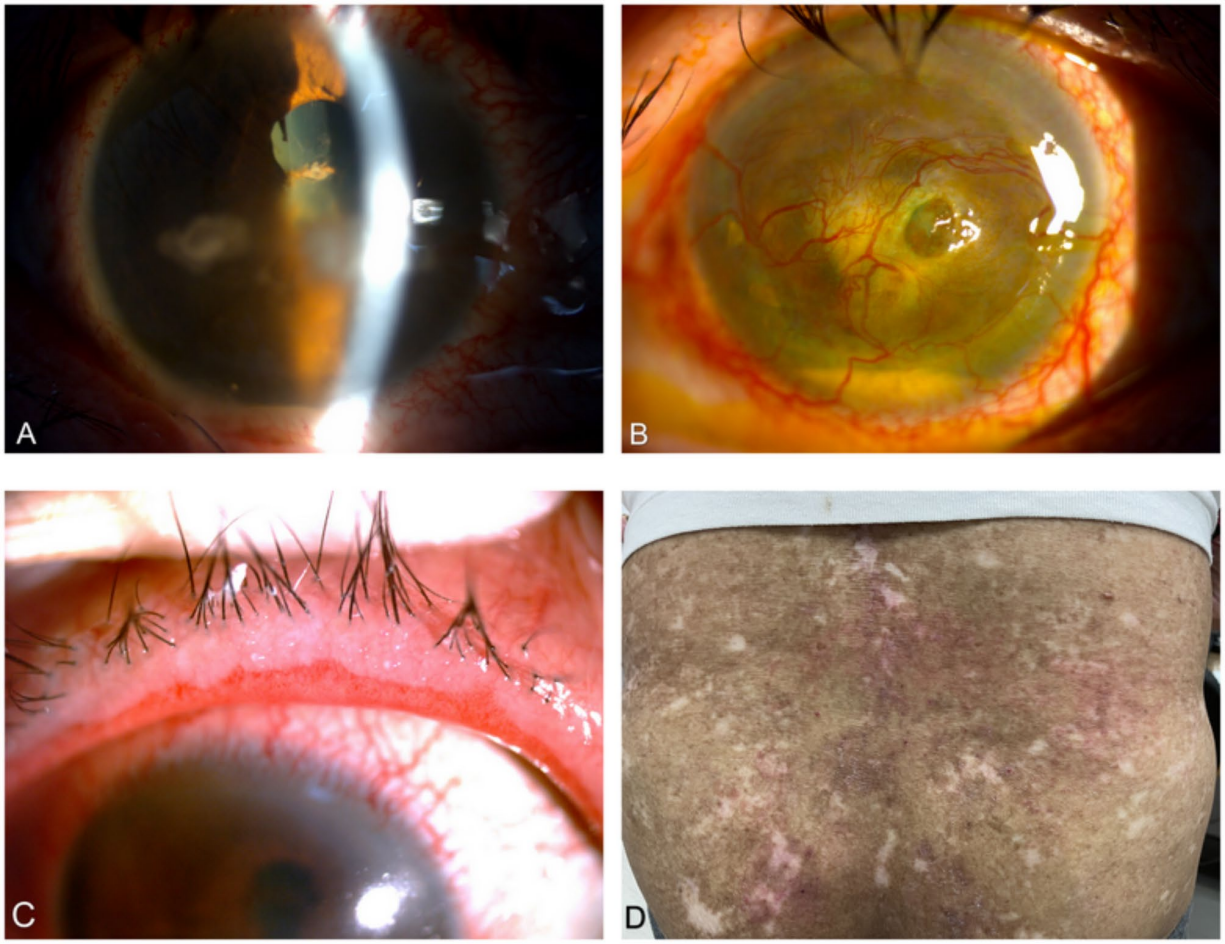
Photography at presentation. (**A**) Image of the right eye (RE) showed prominent anterior chamber inflammation with posterior synechiae. Band keratopathy was due to ocular graft-versus-host disease and keratoconjunctivitis sicca. (**B**) Image of the left eye showed central corneal perforation and hypopyon. (**C**) Eyelid margin erosion of RE. (**D**) Photography of the back showed erythematous skin eruptions

At presentation, his uncorrected visual acuity was 20/200 in the right eye and hand movements in the left eye. Ocular examination showed erythema of the eyelids, telangiectasia of the lid margin, conjunctival hyperemia, superficial punctate keratopathy, prominent anterior chamber non-granulomatous inflammation (cells 4+), posterior synechiae in both eyes, and hypopyon in the left eye (Fig. 1A and B. 1 C). New central corneal perforation with iris incarceration of his left eye was documented. The posterior segment was obscured by the severe posterior synechiae and corneal perforation. The Schirmer test revealed decreased tear secretion of 2 mm in the right eye and 3 mm in the left eye within 5 min. The tear break-up times were 1 and 2 s in the right and left eyes, respectively. Ocular surface staining by fluorescein showed grade 3 in bilateral eyes according to the Oxford grading system. Ultrasonography showed a clear vitreous cavity in both eyes. Physical examination revealed new erythematous skin eruptions over his back (Fig. 1D). He did not report gastrointestinal symptoms.

Blood analysis reported thrombocytopenia, and a slightly elevated erythrocyte sedimentation rate. Human leucocyte antigen (HLA) B27 typing, rheumatoid factor and syphilis serologic tests were negative. Aqueous samples for polymerase chain reaction of herpes simplex virus, varicella zoster virus, and cytomegalovirus were negative.

Treatment with topical 1% prednisolone acetate every 2 h and 1% atropine twice daily to the bilateral eyes, and oral doxycycline 2 times daily were initiated. Lubricants of gel (polyacrylic acid) and ointment (paraffin-based) were applied for dry eye. Additionally, conservative treatment was adopted for the management of the corneal perforation in the left eye. This included the administration of the aforementioned lubricants and topical levofloxacin four times daily. At follow-up 4 weeks later, oral prednisolone 10 mg three times daily and 0.05% topical cyclosporine twice daily were started because of only minimal improvement of the symptoms and signs, and the dosage was tapered gradually according to the clinical condition. In addition, the corneal perforation in the left eye healed with plug-like material formation after 8 weeks of conservative treatment, as well as improvement of dry eye with decreased superficial punctate keratopathy. Intravitreal corticosteroid injection was recommended, but the patient was concerned about the invasive procedure and initially refused. Therefore, to manage the anterior uveitis, a subtenon injection of triamcinolone was performed twice to bilateral eyes, with a dosage of 16 mg (0.4 ml) administered per injection for each eye, which resulted in partial resolution of the anterior chamber inflammation (to cell 1+); however, it rebounded 4 weeks later (to cell 3 + in the right eye and cell trace in the left eye). The patient still refused intravitreal injection; the frequency of topical 1% prednisolone acetate was increased to every hour, and oral prednisolone 10 mg daily was administered over the following 2 months so that the dosage was adjusted based on the ocular condition. Due to progressive blurred vision and persistent anterior uveitis, the patient ultimately had an intravitreal injection of dexamethasone sodium phosphate (0.5 mg) in the right eye, which was administered 6 months after the onset of the initial symptoms. The conjunctival hyperemia and anterior chamber inflammation subsided after only one intravitreal injection. The anterior chamber remained silent, and the topical prednisolone acetate was gradually tapered to once daily. Under relatively stable conditions with a silent anterior chamber, extracapsular cataract extraction was performed 5 months after the intravitreal injection, and penetrating keratoplasty was subsequently performed 9 months after intravitreal injection on the right eye. The last follow-up was conducted 10 months after the intravitreal injection, revealing a visual acuity of 20/200 in the right eye and hand movements in the left eye.

## Discussion and conclusions

We reported a case of simultaneous presentation with ocular GVHD and anterior uveitis following COVID-19 vaccination. Our patient presented with new onset erythema and telangiectasia of the lid margin, conjunctival hyperemia, and superficial punctate keratopathy which are the common signs of ocular GVHD. However, prominent anterior uveitis, which is a very rare (< 1%) ocular manifestation of GVHD, was also observed [7].

Both uveitis and GVHD have been reported respectively after vaccination against severe acute respiratory syndrome coronavirus 2 (SARS-CoV-2) [5, 6]. Nevertheless, the simultaneous occurrence of ocular GVHD and anterior uveitis in recipients of HSCT after COVID-19 vaccination is rare and has not been extensively discussed in previous literature.

Recipients of HSCT are at risk of infection from various pathogens and therefore vaccination is a crucial protection for these patients. The immune response generated by vaccination in patients with HSCT is usually weaker than that in healthy individuals during the initial months or years after transplantation and gradually improves over the next 2–3 years [8]. As a result of the COVID-19 pandemic, novel vaccines based on mRNA techniques, including the Pfizer-BioNTech BNT162b2 vaccine and Moderna mRNA-1273 vaccine were developed. A previous publication reported an increasing incidence of GVHD following COVID-19 vaccination in allo-HSCT recipients, particularly in patients with prior GVHD and recent transplant.

The mechanism between vaccination and the occurrence of GVHD is not well established. Molecular mechanisms of inflammatory triggers in GVHD include sterile damage associated molecular patterns (DAMPs) molecules and pathogen-associated molecular patterns (PAMPs). The former implies that molecules are released into the extracellular space where tissue damage and immune activation can be stimulated. The latter involves microbes such as bacteria and viruses [9]. Immunological mechanisms of mRNA vaccines and DAMPs/PAMPs have also been discussed. Cytokines can be induced by DAMPs/PAMPs and result in reactogenicity and immunogenicity due to vaccination. The components of vaccines may serve as PAMPs/DAMPs, which may trigger the immune response and subsequently lead to GVHD [10].

In addition, limited studies on the association between vaccination against SARS-CoV-2 and the occurrence of GVHD have been reported. Dat Ngo et al. revealed that the incidence of new and worsening GVHD was 15.4% for BNT162b2 and 11.8% for mRNA-1273 (*p* = 0.491) after receiving SARS-CoV-2 vaccination. Among these cases, the ocular presentation accounted for approximately 20%. Previous chronic GVHD and transplant dates less than 180 days were associated to a higher rate of GVHD [5]. Andrew et al. reported that in allogeneic HSCT recipients with chronic GVHD, the occurrence of symptom exacerbation following COVID-19 vaccination was relatively common (26.5%) [11]. Ali, H., et al. reported the incidence of adverse events following the Pfizer-BioNTech BNT162b2 vaccine and Moderna mRNA-1273 vaccine [12]. Among the 113 patients, worsening of chronic GVHD occurred in 3.5%, and new chronic GVHD occurred in 9.7%. Only 3 of the 13 patient with GVHD had ocular symptoms and signs, which were treated with either systemic prednisone, tacrolimus, sirolimus or prednisolone 1% eye drops and achieved a well-controlled or a resolved disease status.

With regard to uveitis, Charlotte A. et al. reported the rates of uveitis reactivation associated with COVID-19 vaccination in a large-scale population with previous uveitis. The study revealed an increased risk of uveitis after the first dose of COVID-19 vaccination. Among the cases, the majority were attributed to idiopathic etiology and anterior uveitis was most common [6]. Rabinovitch, T., et al. reported 21 anterior uveitis cases following Pfizer-BioNTech BNT162b2 vaccination, which resolved after treatment with topical corticosteroid [13]. In addition, the aforementioned case series did not include recipients of HSCT, which may have contributed to the different clinical presentations. The special immune system of recipients of HSCT may contribute to the completely different manifestation in our case. After two subtenon steroid injections, the uveitis in our case didn’t subside until intravitreal corticosteroid injection 6 months after vaccination. Intravitreous steroid injection is considered to be effective and important in refractory or chronic uveitis. Other management options for uveitis, such as pulse therapy with intravenous steroid injection, were not performed due to concerns about potential systemic side effects and the potential impact on vaccine effectiveness during the COVID-19 pandemic. Additionally, intracameral steroid injection was not chosen for the following reasons: (1) the formation of pseudohypopyon after intracameral injection could complicate clinical judgment for the physician, and (2) intravitreal injection could be performed in the outpatient department, while intracameral injection required arrangements in the operating room, which the patient declined. On the other hand, the humoral response diminished progressively 4–6 months after COVID-19 vaccination, which was indicated by previous systemic review [14]. The resolution of uveitis in our case occurred 6 months after vaccination, which might have contributed to either intravitreous steroid injection or a diminished immune response. Further investigation is required to determine whether anterior uveitis is a manifestation of ocular GVHD or an independent manifestation after vaccination.

There are certain limitations in this report. First, the uveitis subsided shortly after the intravitreal steroid injection, approximately 6 months after the onset of symptoms. The observed temporal relationship suggests the efficacy of the intravitreal steroid; however, as discussed earlier, the regression of uveitis could possibly be attributed to the diminished immune response following vaccination. Hence, we cannot completely exclude the possibility that the uveitis resolved due to the progressively weakened immune response. Second, after consulting with the hematologist, systemic immunomodulators such as methotrexate and cyclosporine were not administered to the patient due to concerns about his hematologic condition. This limitation restricts the available treatment options in such circumstances.

Clinicians should be alert to the possibility of acute exacerbation of ocular GVHD and anterior uveitis following vaccination against SARS-CoV-2 in patients who have undergone allogeneic HSCT. Under the condition that immunogenicity could be different in this group of patients, disease activity may be recurrent and refractory, and a more chronic course should be expected. Early diagnosis, close monitoring, and aggressive treatment, including intravitreal corticosteroid injection, in addition to topical or systemic steroids in usual cases, should be considered due to the possibility of severe complications. We did not use systemic immunomodulatory therapy because of the risk of infection and attenuation of the vaccination. Further studies and case reports are required to determine the role of systemic immunomodulatory therapy in patients with anterior uveitis and ocular GVHD following COVID-19 vaccination.

## Data Availability

All relevant findings are contained within this manuscript.

## List of abbreviations

COVID-19: Coronavirus disease 2019
DAMP: Damage-associated molecular pattern
GVHD: Graft-versus-host disease
HSCT: Hematopoietic stem cell transplantation
PAMP: Pathogen-associated molecular pattern
SARS-CoV-2: Severe acute respiratory syndrome coronavirus 2
